# Efficient, Vpx independent shRNA transduction of monocyte derived dendritic cells as a tool for studying HIV transmission to primary T-cells

**DOI:** 10.1186/1742-4690-10-S1-P100

**Published:** 2013-09-19

**Authors:** Wojciech Witkowski, Veronica Iannucci, Evelien Naessens, Hanne Vanderstraeten, Bruno Verhasselt

**Affiliations:** 1 Department of Clinical Chemistry, Microbiology and Immunology, Ghent University, Ghent, Belgium

## Background

Dendritic cells present at mucosal sites are considered initial targets for HIV transmission. Due to SAMHD1 expression, they are highly resistant to HIV infection themselves, but act as shuttles for viral transmission to T cells. Knock-down of HIV host cellular partners is a powerful method in HIV research. At present, lentiviral shRNA delivery to Monocyte Derived Dendritic Cells (MDDC) requires the use of virus-like particles carrying the Vpx protein in order to alleviate SAMHD1 mediated restriction. Such an approach results in the desired high transduction efficiency, but simultaneously abrogates resistance to HIV thus allowing for viral replication which might obscure the actual outcomes of MDDC-HIV interactions. We present a method for efficient shRNA transduction of MDDC for subsequent studies of HIV transmission to CD4+ T-cells, without the need for Vpx.

## Methods

MDDC were obtained by culturing primary monocytes for 6 days in presence of lL-4 and GM-CSF. At day 1 after monocyte isolation, cells were transduced with shRNA encoding lentiviral vectors (Sigma MISSION^®^) at MOI of 1 (titration on HEK293T cell line) by addition of polybrene and using spinoculation. Transduction efficiency was determined by measuring the fraction of eGFP expression (encoded by the lentiviral vector) by flow cytometry. At day 5 post transduction, cells were exposed to NL4.3 HIV virus harboring a marker gene. Twenty four hours later the cells were extensively washed with culture medium to remove unbound virus and autologous, primary CD4+ T cells were added at 1:2 ratio. Infection of T-cells in MDDC-T-cell cocultures was subsequently monitored by flow cytometry to detect marker gene expressing cells in low scatter and/or CD3+ population.

## Results

Efficient transduction of MDDC (≈95%) as judged by eGFP expression was obtained. This high efficiency avoids puromycin selection and therefore simplifies the protocol while preventing possible side effects. Notably, the transduced MDDC did not show a marked increase in CD80, CD83 and CD86 expression levels. Transduced MDDC are able to capture and transmit the virus to T cells. We have observed a positive correlation between shRNA mediated downregulation of certain MDDC surface markers and transmission of HIV to CD4+ T cells as compared to cells transduced with a control vector encoding a scrambled shRNA sequence not known to target any human gene.

## Conclusions

We have established a method for lentiviral encoded shRNA transductions allowing for studies of MDDC cellular factors involved in HIV transmission to CD4+ T cells without the need to alleviate SAMHD1 induced restriction. Transduced MDDC remain immature as judged by expression of CD80, CD83 and CD86 cell surface receptors. Preliminary results provide validation of the approach by positively correlating downregulation of MDDC surface markers with trans-infection of CD4+ T cells.

